# Mechanisms Underlying the Benefits of Coffee in Postoperative Ileus

**DOI:** 10.3390/nu18152503

**Published:** 2026-08-03

**Authors:** Ke Zhang, John C. Johnson, Sena Saygili, Daniel W. Shi, Neeraja Recharla, Ramasatyaveni Geesala, Xuan-Zheng Shi

**Affiliations:** 1 Department of Internal Medicine, Division of Gastroenterology, The University of Texas Medical Branch, Galveston, TX 77555-0655, USA; kezhang@utmb.edu (K.Z.); nerechar@utmb.edu (N.R.); rageesal@utmb.edu (R.G.); 2 John Sealy School of Medicine, The University of Texas Medical Branch, Galveston, TX 77555-0655, USA; john.johnson@bcm.edu (J.C.J.); sesaygil@utmb.edu (S.S.); dwshi@utmb.edu (D.W.S.)

**Keywords:** coffee, postoperative ileus, intestinal motility, inflammation

## Abstract

Background and Aims: Postoperative ileus (POI) is a motility dysfunction associated with inflammation in the gastrointestinal (GI) tract after abdominal surgery. Management relies on supportive care, as there is no effective medical treatment. Clinical trials found that coffee consumption improves bowel movement and shortens hospital stays in POI. We aimed to investigate the mechanisms underlying the beneficial effect of coffee in an animal model of POI. Methods: Mouse POI was established by manipulation of the small intestine with wet swab applicators for 5 min. Mice were then treated with water, regular or decaffeinated coffee (10 mg per day) by oral gavage and euthanized 24 h after the operation. Key Results: Intestinal manipulation slowed GI transit rate from 5.64 ± 0.47 to 3.77 ± 0.16 (N = 6, measured by geometric center), reduced intestinal muscle contractility, and induced an acute inflammatory response with increased expression of proinflammatory mediators such as IL-6, IL-1, CCL2, and CXCL-1 in the POI intestine. Coffee treatment (regular or decaffeinated) did not reduce inflammation or the expression of inflammatory mediators but significantly improved muscle contractility and increased GI transit to 4.59 ± 0.31 (N = 5) and 4.80 ± 0.29 (N = 5) in POI mice (regular and decaffeinated coffee, respectively). Regular or decaffeinated coffee dose-dependently (0.1–10 mg/mL) increased contractility of intestinal muscle strips. The contractile effect was not affected by neural toxin tetrodotoxin (10^−6^ M) or cholinergic nicotinic antagonist hexamethonium (10^−4^ M) but was completely abolished by muscarinic receptor antagonist atropine (10^−6^ M). Conclusions: Coffee consumption does not attenuate inflammatory response but improves GI motor function and stimulates intestinal smooth muscle contractions in the mouse model of POI. Coffee stimulates contractions in a caffeine-independent manner through a cholinergic muscarinic receptor-dependent mechanism.

## 1. Introduction

Postoperative ileus (POI) is a common complication of abdominal surgery, characterized by impaired motility associated with inflammatory response in the gastrointestinal (GI) tract [1]. Clinically, POI manifests as abdominal distension, nausea, vomiting, constipation, and intolerance of oral intake [2,3]. POI is a major healthcare burden as it increases hospital stays, cost of care, and 30-day readmission rates [3,4]. Up to 30% of patients develop POI after abdominal surgery [3,4]. A recent systematic meta-analysis reported that POI raises total hospital costs by 66% among patients going through abdominal surgery [4].

Unfortunately, it is unclear how abdominal surgery or even surgeries in other parts of the body, e.g., knees or limbs, may lead to POI. The current understanding is that neurogenic and pharmacological factors may contribute to motility dysfunctions in the early phase of POI (i.e., the first few hours after operations), whereas intestinal inflammatory response is the key mechanism underlying POI, especially in the late phase, which may last for days [1,4,5]. Inflammation leads to motility dysfunction by affecting the enteric nervous system and smooth muscle function [6]. However, the mechanisms of inflammation and motility dysfunction in POI are incompletely understood. Consequently, there is no effective medical treatment for POI, and the current management plan relies on supportive care.

Recent years have seen growing interest in interventions to accelerate postoperative recovery. Among these, coffee has emerged as a promising, low-cost strategy. Clinical evidence suggests that coffee consumption improves bowel movement, reduces the incidence of postoperative ileus and shortens the duration of hospital stay in postoperative patients [7,8,9]. In a randomized clinical trial, Muller et al. first reported that coffee consumption after colectomy was safe and associated with a reduced time to first bowel movement [7]. Dulskas et al. found that decaffeinated coffee also reduced the time to first bowel movement and shortened the time to tolerance of solid food and to the first flatus [8]. Their findings suggest that the beneficial effects of coffee on gastrointestinal recovery are not solely dependent on caffeine content. Watanabe et al. conducted a meta-analysis regarding the effect of coffee consumption on postoperative ileus, including 13 trials and 9 ongoing trials. The analysis further indicated that coffee consumption, regardless of its caffeine content, was effective across diverse types of abdominal surgeries, reinforcing its generalizability as a postoperative intervention [9]. These findings highlight coffee’s potential benefits in POI. Elucidating the underlying mechanisms of coffee’s benefits in POI may inform novel therapeutic approaches for POI and other motility disorders such as constipation and irritable bowel syndrome [10,11].

In the present study, we conducted a series of experiments in a mouse model of POI to assess the hypothesis that coffee consumption is beneficial in POI by attenuating inflammation and/or enhancing GI motor activity. We determined the GI transit, smooth muscle contractility, inflammation score, and representative inflammatory mediators (e.g., IL-1beta, IL-6) and chemokines (e.g., CCL2 and CXCL-1) at 24 h after an operation in sham and POI groups treated with vehicle, regular or decaffeinated coffee. The 24 h time point was selected, as previous work [5,6] and our pilot data demonstrated that motility is severely compromised and inflammatory response nears its peak at 24 h following the operation in the model. First, we determined whether treatment with regular or decaffeinated coffee solution reduced inflammation and decreased the expression of proinflammatory mediators. We then investigated whether coffee treatment improved GI transit and smooth muscle contractility in POI. We aimed to further investigate the mechanisms involved in the beneficial effects of coffee on inflammation and/or motility, if any.

## 2. Materials and Methods

### 2.1. Mouse Model of Postoperative Ileus

C57BL/6J mice (20–25 g; both male and female) were obtained from Jackson Laboratory and used in all the experiments. Mice were maintained under standard housing conditions (22 °C, 12 h light/dark cycle) with unrestricted access to food and water. All animal procedures were approved by the Institutional Animal Care and Use Committee of the University of Texas Medical Branch and were conducted in accordance with the National Institutes of Health Guide for the Care and Use of Laboratory Animals.

A murine model of postoperative ileus (POI) was established as previously described [12,13]. Briefly, mice were anesthetized with isoflurane, and a midline laparotomy was then performed. The small intestine was gently exteriorized onto sterile saline-moistened gauze while the stomach, cecum, and colon remained within the abdominal cavity. Intestinal manipulation was performed with two saline-moistened cotton swabs through gentle compression and rolling of the entire small intestine, extending from the duodenum to the cecum, for 5 min. The bowel was then carefully returned to the abdominal cavity, and the incision was closed in two layers [12,13]. Sham-operated mice underwent laparotomy for the same duration without intestinal manipulation.

To allocate animals to groups, we followed a randomization process. In brief, mice were blindly numbered, and the even and odd numbers were allocated to the sham and POI groups, respectively. Based on power analysis (nQuery), we used at least 5 animals (up to 6) in each experimental group for the in vivo studies. We aimed for equal numbers of males and females in each group, or 3 males/2 females when the N was 5 in a group.

### 2.2. In Vivo Coffee Treatment Protocol

Regular and decaffeinated coffee were purchased from Starbucks Corporation (Seattle, WA, USA). According to the manufacturer, 97% or more of caffeine had been removed in the decaffeinated coffee. The regular and decaffeinated coffee solutions were prepared by brewing the 100% Arabica coffee or decaffeinated coffee powder in hot water at a 14 mL of water per 1 g of coffee powder ratio. The brewed solutions were then centrifuged and filtered as previously described [14,15]. Sham-operated and POI mice were randomly assigned to receive either vehicle control (water; Ctr), regular coffee (Coff), or decaffeinated coffee (Decaf). All treatments were administered by oral gavage with a volume of 200 µL [15,16]. Mice received three doses at 1, 6, and 22 h after the surgical procedure. Coffee solutions were prepared to deliver a total dose of 10 mg within the 24 h treatment period (e.g., 3.33 mg per dose).

### 2.3. Gastrointestinal Transit Measurements

Gastrointestinal transit function was evaluated using the distribution of phenol red in an oral methylcellulose meal (1.5% methylcellulose containing 0.125 mg of non-absorbable phenol red) within the intestine [17,18]. Ninety minutes before euthanasia, mice received 250 μL of phenol red methylcellulose mixture by oral gavage. Following euthanasia, the entire gastrointestinal tract, from the stomach to the distal colon, was removed. Gastric contents, the small intestine (divided into 8 equal-length segments), cecum and colon (considered a single segment) were collected separately. The collected tissues were immersed in 0.1 N NaOH solution and homogenized. One milliliter of the homogenate supernatant was mixed with 0.1 mL of 20% (*v*/*v*) trichloroacetic acid (TCA) solution to precipitate proteins. After centrifugation (8000 rpm, 5 min), 0.5 mL of the supernatant was combined with 0.5 N NaOH. The optical density (OD) value was read at 560 nm to quantify the phenol red recovery within each intestinal segment. Gastrointestinal transit was expressed as the geometric center (GC), calculated using the formula Σ (phenol red counts per segment × segment number), enabling quantitative statistical comparisons between experimental groups [18,19].

### 2.4. Intestinal Smooth Muscle Contractility Studies

Intestinal tissue located 10–20 cm proximal to the ileocecum junction was collected immediately after euthanasia and transferred to carbogenated Krebs solution (mmol/L: 2.5 CaCl_2_, 5.9 KCl, 1.2 MgCl_2_, 15.4 NaHCO_3_, 1.5 NaH_2_PO_4_, 120.3 NaCl, and 11.5 D-glucose) [15,19,20,21]. The ileal tissue was opened along the mesenteric border and placed in a Sylgard-lined Petri dish containing Krebs buffer. Full-thickness ileal strips (2 × 10 mm) were cut along the longitudinal axis and secured within individual muscle baths (Radnoti Glass, Monrovia, CA, USA) containing 10 mL of carbonized Krebs solution at 37 °C. Contractile activity was recorded using an isometric force transducer (Grass Instruments, West Warwick, RI, USA) coupled to a Biopac data acquisition system (Biopac Systems, Goleta, CA, USA) as previously described [20,21]. Smooth muscle contractility was measured after equilibration for 60 min under a resting tension of 1 g. The contractility of the muscles was measured following exposure to different concentrations of acetylcholine (ACh) (10^−7^ to 10^−3^ M). Responses at each concentration were recorded for 2 min. The area under the contraction curve (AUC) was calculated after subtracting baseline activity.

To evaluate the direct effects of coffee, the ileal muscle strips were exposed to increasing concentrations of regular or decaffeinated coffee (0.1–10 mg/mL). The contractile response to each concentration was recorded for 2 min. To investigate the underlying mechanism, the responses of the muscles to regular or decaffeinated coffee (5 mg/mL) was assessed in the presence or absence of hexamethonium (Hex, 10^−4^ M), a nicotinic cholinergic receptor antagonist; atropine (Atr, 10^−6^ M), a muscarinic receptor antagonist; or tetrodotoxin (TTX, 10^−6^ M), a voltage-gated sodium channel blocker that inhibits neuronal activity [15,22,23]. For the in vitro study of coffee’s effects in muscle strips, we performed 4 independent experiments. In each experiment, the tissues were taken from different naïve mice. We aimed for equal numbers of male and female animals in each group.

### 2.5. Histological Studies

Full-thickness ileal tissue samples from mice in different experimental groups were collected, fixed in formalin, embedded in paraffin, and processed as previously described. Sections were stained with hematoxylin and eosin at the University of Texas Medical School Histopathology Core. Stained sections were viewed using a Revolve microscope from Echo Labs (Echo, San Diego, CA, USA) [24]. Intestinal inflammation was graded using a previously published semiquantitative scoring system based on mucosal injury, edema, and increased inflammatory cell infiltration, with scores ranging from 0 (normal) to 3 (severe) [13,25]. Two independent investigators scored each specimen blindly, and their average reading served as the final score.

### 2.6. RNA Extraction and Quantitative RT-PCR

Total RNA was isolated from mouse ileal tissue using the RNeasy kit (Qiagen, Valencia, CA, USA). Two micrograms of RNA were reverse transcribed with the SuperScript III First-Strand Synthesis System (Invitrogen, Carlsbad, CA, USA) [25,26,27]. Quantitative real-time PCR was performed using a Bio-Rad CFX96 Real-Time PCR system (Hercules, CA, USA) as previously described [28,29]. TaqMan probes specific for mouse IL-1β (Mm00434228_m1), 1L-6 (Mm00446190_m1), CCL2 (Mm00441242_m1), and CXCL-1 (Mm04207460_m1) were obtained from Invitrogen. Relative gene expression was determined using the comparative Ct (ΔΔCt) method, with 18S rRNA (Part no. 4352930E, Applied Biosystems) serving as the endogenous reference gene.

### 2.7. Statistical Analysis

Data points are expressed as means ± SEM, unless otherwise specified. Each data point is the average of two measures for the histology, contractile response, and quantitative PCR studies. GraphPad Prism version 10 was used for statistical analyses. For comparisons of multiple groups, statistical analysis was performed by analysis of variance (ANOVA) with two-way repeated measures followed by Tukey’s test in post hoc analysis, or by Dunn’s test if data in a group did not follow normal distribution. Student’s *t*-test was used for comparisons of two groups. A *p* value of ≤0.05 was considered statistically significant [28,29].

## 3. Results

### 3.1. Effects of Coffee and Decaffeinated Coffee on Inflammatory Response in POI

**Figure 1:**
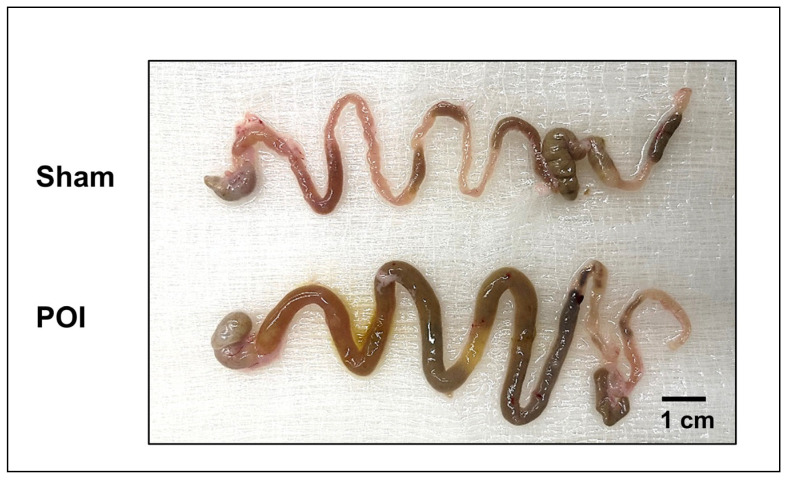
**Mouse model of postoperative ileus (POI).** The mouse model of POI was established by manipulation of the small intestine with wet swab applicators for 5 min. Intestinal manipulation (IM) led to food retention, gut inflammation, and lumen distention in POI mice. Sham control mice underwent laparotomy but no manipulation. Mice were euthanized 24 h post-operation. Black bar = 1 cm. The image is representative of 6 independent experiments.

Compared to the sham operation, intestinal manipulation (IM) induced pronounced histological, inflammatory, and functional alterations in the small intestine in our model. We found that the small intestines of the manipulated mice (POI) were significantly distended and inflamed, with accumulated food chyme, compared to sham mice (Figure 1).

**Figure 2:**
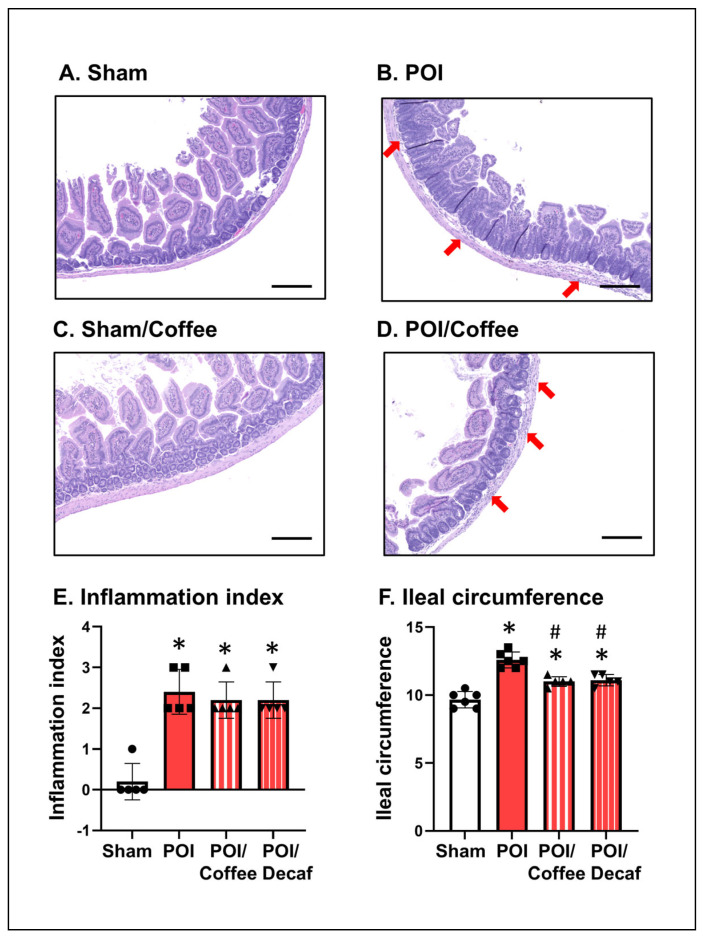
**Effects of caffeinated (Coffee) and decaffeinated coffee (Decaf) on gut inflammation in POI.** (**A**–**D**) Representative microscopic images of the intestinal specimens in mice with sham operation (**A**), POI (**B**), and sham with coffee treatment (**C**) and POI with coffee treatment (**D**). Arrows point to inflammatory infiltrates. Bar = 200 µm. (**E**) Summary of intestinal inflammation index in groups of sham, POI, POI treated with coffee, and POI with decaffeinated coffee. (**F**) Summary of maximal intestinal circumference in groups of sham, POI, POI treated with coffee, and POI with decaffeinated coffee. N = 5 mice in each group. * *p* < 0.05 vs. sham. ^#^ *p* < 0.05 vs. POI.

Microscopic scanning revealed that there were disrupted epithelium, edema, and increased inflammatory infiltrations involving the muscularis externa in the intestine of POI mice (Figure 2A–D). The microscopic inflammation index was 2.2 ± 0.37 in POI mice and 0 (*p* < 0.05, N = 5) in sham controls (N = 5) (Figure 2E). Treatment with caffeinated (coffee) or decaffeinated coffee did not significantly reduce inflammation index in the POI mice (2.0 ± 0.32 and 2.0 ± 0.45, *p* > 0.05 vs. POI control) (Figure 2E). The circumference of the intestine was 12.4 ± 0.5 mm in the POI group, which is significantly greater than the sham group (10.1 ± 0.3 mm, *p* < 0.05). Interestingly, treatment with coffee or decaffeinated coffee significantly reduced the intestinal circumference in the POI mice (Figure 2F).

**Figure 3:**
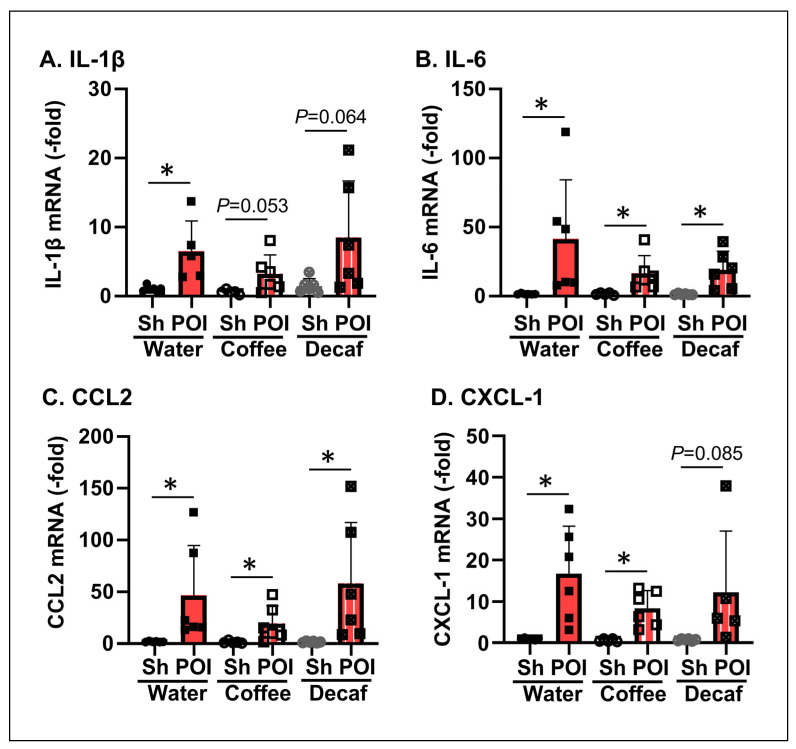
**Effects of caffeinated and decaffeinated coffee on mRNA expressions of proinflammatory cytokines and chemokines in POI.** The mRNA expressions of IL-1β (**A**), IL-6 (**B**), CCL-2 (**C**), and CXCL1 (**D**) were significantly increased in the small intestine of POI mice. Treatments with caffeinated (coffee) or decaffeinated coffee (decaf) did not significantly reduce the expressions of cytokines and chemokines in POI mice. N = 5 or 6. * *p* < 0.05 vs. sham of the treatment groups.

We also determined the expression of proinflammatory cytokines and chemokines in sham and POI mice with and without treatments of coffee and decaffeinated coffee. Quantitative RT-PCR measurements showed that mRNA levels for IL-1β, IL-6, CCL2, and CXCL-1 in the intestine were all significantly increased in POI mice compared with sham mice (N = 5 or 6, * *p* < 0.05) (Figure 3A–D). Coffee and decaffeinated coffee did not significantly reduce the expression of the inflammatory mediators in POI mice (*p* > 0.05) (Figure 3A–D).

### 3.2. Effects of Coffee and Decaffeinated Coffee on Gastrointestinal Transit in POI

**Figure 4:**
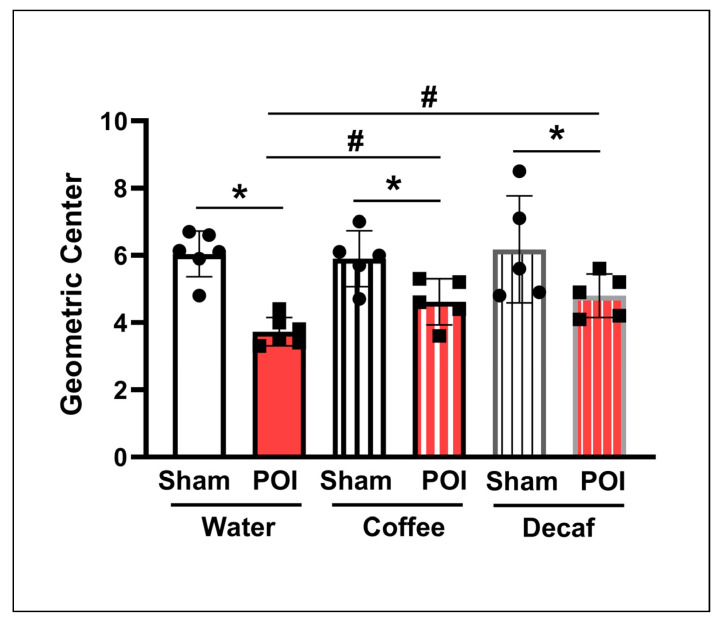
**Effects of caffeinated and decaffeinated coffee on gastrointestinal transit in POI.** Sham and POI mice were treated with vehicle (water), coffee, or decaffeinated coffee, respectively, and euthanized 24 h after operation for the determination of geometric center as described in Methods. N = 5 or 6. * *p* < 0.05 vs. sham in the treatment group. ^#^ *p* < 0.05 vs. POI in water treatment group.

We then determined if coffee treatment had any beneficial effect on GI transit in sham and POI mice. Compared with the sham group, the GI transit rate, measured by geometric center (GC), was significantly slowed in POI (5.64 ± 0.47 vs. 3.77 ± 0.16 for sham and POI, respectively, *p* < 0.05, N = 6). Importantly, treatment with coffee did not affect GI transit in sham controls but significantly improved transit in POI mice (5.88 ± 0.47 vs. 4.59 ± 0.31, *p* < 0.05, N = 5) (Figure 4). Decaffeinated coffee had similar benefits on transit as coffee (6.18 ± 0.73 vs. 4.80 ± 0.29, *p* < 0.05, N = 5) (Figure 4).

### 3.3. Effects of Coffee and Decaffeinated Coffee on Intestinal Muscle Contractility in Mice with POI

**Figure 5:**
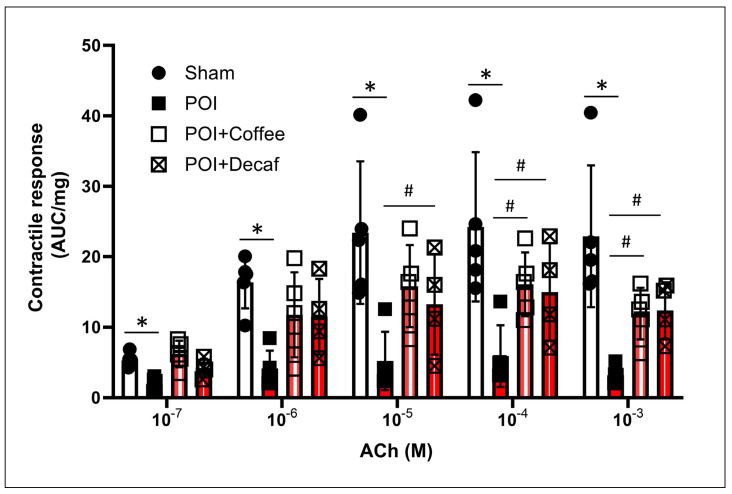
**Effects of caffeinated and decaffeinated coffee on intestinal smooth muscle contractility in POI.** After operation, sham and POI mice were treated with vehicle (water) or coffee, or decaffeinated coffee, respectively. Mice were euthanized 24 h after operation. Ileal muscle strips were isolated along the longitudinal muscle axis, and the smooth muscle contractile responses to acetylcholine (ACh 10^−7^ to 10^−3^ M) were determined in a 10 mL muscle bath. N = 4 or 5 mice per group. * *p* < 0.05 between sham and POI. ^#^ *p* < 0.05 between POI and POI/Coffee or POI/Decaf.

To investigate whether coffee treatment has any genomic effects on smooth muscle function, we conducted a muscle bath study to compare the contractile response of ileal smooth muscle to acetylcholine (ACh, 10^−7^ to 10^−3^ M) in sham and POI mice treated with oral administration of vehicle, coffee, and decaffeinated coffee. We detected a dramatically reduced smooth muscle contractile response to ACh in the POI tissue, compared with sham. However, administration of regular or decaffeinated coffee significantly improved the intestinal muscle contractility in POI mice (Figure 5).

### 3.4. Direct Effects of Coffee and Decaffeinated Coffee on Ileal Smooth Muscle Contractions

**Figure 6:**
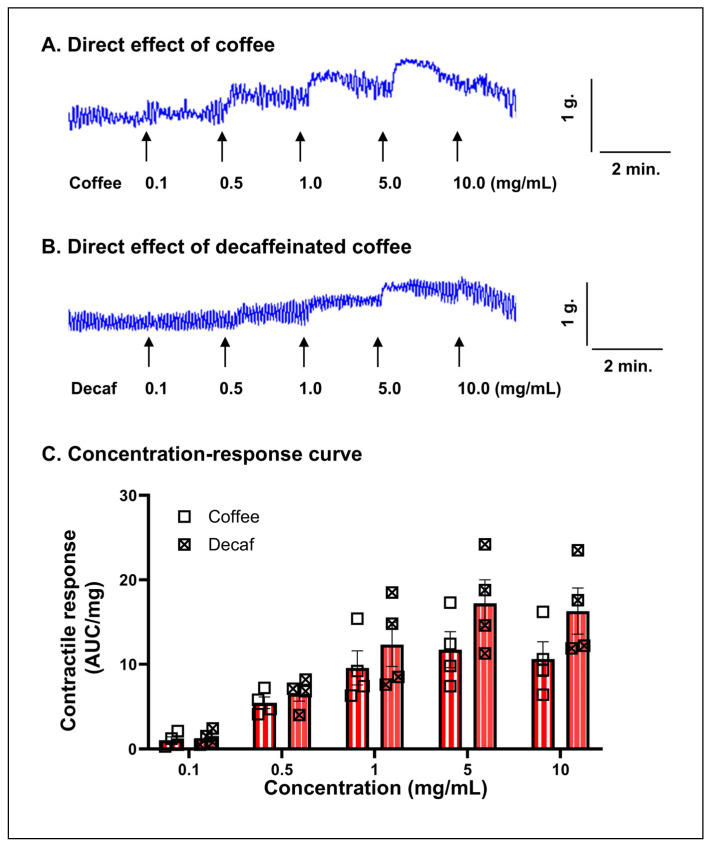
**Effects of caffeinated and decaffeinated coffee on ileal muscle contractions in vitro.** Ileal muscle strips were isolated from sham mice, and their longitudinal muscle contractility was recorded in a muscle bath. The direct effects of regular coffee (**A**) and decaffeinated coffee (Decaf, (**B**)) at different concentrations (0.1–10 mg/mL) on muscle contractility were measured in the first 2 min after the addition of each dose. The effects are summarized in (**C**). ↑ points to the time each indicated dose of coffee or decaf was added to the muscle bath. N = 4 independent experiments.

Next, we studied the in vitro effect of coffee on ileal smooth muscle strips in the muscle bath studies. Coffee treatment in vitro induced a robust contractile response of ileal smooth muscle in a dose-dependent manner (0.1 to 10 mg/mL) (Figure 6A). Decaffeinated coffee (0.1 to 10 mg/mL) also increased smooth muscle contractility to a similar extent as regular coffee (Figure 6B,C).

### 3.5. Role of Cholinergic Muscarinic Receptors in Mediating the Contractile Effects of Coffee

**Figure 7:**
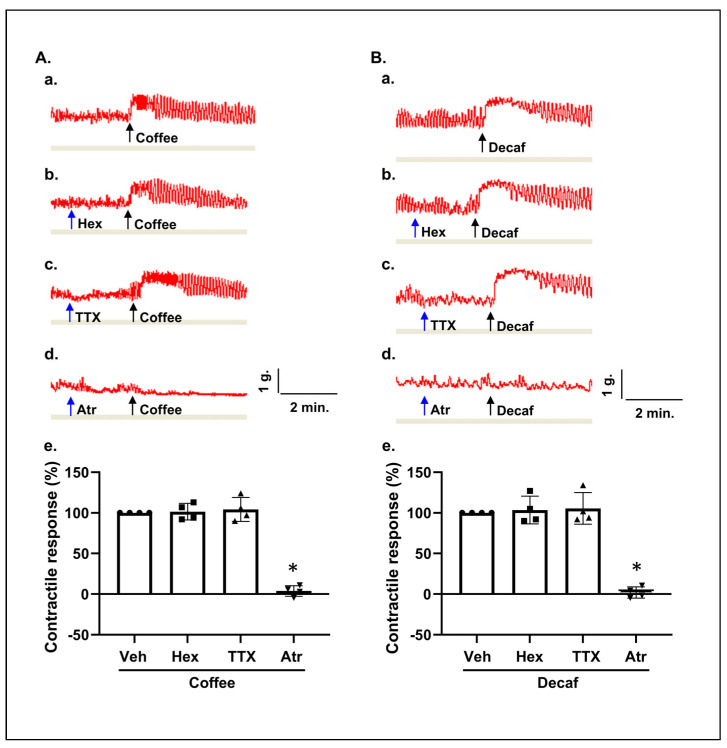
**Site of action of caffeinated and decaffeinated coffee on mouse intestinal smooth muscle contractions.** Intestinal muscle strips were isolated from sham mice, and their longitudinal muscle contractile activity was recorded in a muscle bath. (**A**) Contractile responses to 5 mg/mL regular coffee (Coffee) were recorded in the absence (**a**) and presence of nicotinic receptor antagonist hexamethonium (Hex, 10^−4^ M) (**b**), neurotoxin tetrodotoxin (TTX, 10^−6^ M) (**c**), and muscarinic receptor antagonist atropine (Atr, 10^−6^ M) (**d**). (**B**) Contractile responses to 5 mg/mL decaffeinated coffee (Decaf) were recorded in the absence (**a**) and presence of Hex 10^−4^ M (**b**), TTX, 10^−6^ M (**c**), and Atr, 10^−6^ M (**d**). The effects of Hex, TTX, and Atr on the contractile activities induced by caffeinated or decaffeinated coffee were summarized in (**A**(**e**)) and (**B**(**e**)), respectively. ↑ points to the time each indicated drug was added to the muscle bath. N = 4 independent experiments. * *p* < 0.05 between Veh and Atr groups.

Finally, we aimed to determine the site(s) of action of coffee on intestinal smooth muscle contractions (Figure 7). When cholinergic nicotinic receptors were blocked with hexamethonium (10^−4^ M) or neural activity was inhibited with tetrodotoxin (TTX, 10^−6^ M), the contractile effects of coffee or decaffeinated coffee remained intact in the muscle strips (Figure 7A,B). However, the contractile response of coffee or decaffeinated coffee was completely attenuated in the presence of atropine (10^−6^ M), a cholinergic muscarinic receptor antagonist (Figure 7A,B).

## 4. Discussion

Clinical studies have shown that coffee intake improves bowel movement, reduces the incidence of ileus, and shortens the duration of hospital stay among postoperative patients [7,8,9]. However, the mechanisms of the beneficial effects were not clear. In the present study, we found that intestinal manipulation led to slowed GI transit and significant inflammatory response in the mouse model of POI. POI mice presented increased inflammatory infiltrations and production of cytokines, chemokines, and other proinflammatory molecules in the intestinal tissues. Coffee treatment significantly improved GI transit in mice with intestinal manipulation, confirming the beneficial effect of coffee in POI. While impaired motility with slowed GI transit is the hallmark of POI, gut inflammation and smooth muscle dysfunction are considered to contribute to motility dysfunction in POI [4,5]. We thus determined if the benefits of coffee in POI are through its inhibition of gut inflammation or improvement of smooth muscle contractile function. Our results showed that coffee treatment did not significantly reduce inflammation scores or the expression of the selected inflammatory mediators in POI at 24 h after operation. However, it significantly improved GI transit and intestinal smooth muscle contractility. Thus, our study in the mouse model suggests that coffee benefits POI possibly by improving motor activity and augmenting smooth muscle contractility rather than reducing inflammation.

In the studies to determine the effect of coffee in postoperative state in humans, patients were given 3~4 cups of coffee daily (three times) [7]. In our protocol, coffee was administered at 10 mg in 24 h in three doses (1, 6, and 22 h after operation). For mice undergoing GI transit study, the dye was injected into the stomach two hours after the last dose of coffee. This design allowed us to measure the overall but not the immediate effect of coffee on GI transit. We found that coffee administration significantly improved GI transit in POI mice but not in sham mice, confirming the efficacy of coffee in POI. We believe that the improved transit in POI is associated with enhanced smooth muscle contractile function. As coffee treatment directly stimulated smooth muscle contractions (shown in our in vitro study), the coffee-stimulated contractile activities would help propel intraluminal contents and decrease the mechanical stress in the intestine. In fact, we found that the intestinal circumference, a measurement of mechanical stress in the lumen, decreased significantly in coffee-treated POI mice compared to POI controls. A decrease in mechanical stress of the intestine would further improve smooth muscle contractility and gut motor function. As it is demonstrated elsewhere [18,21], increased mechanical stress in a distended intestine is associated with production of mechanoresponsive proinflammatory molecules and prostaglandins, which lead to suppressed smooth muscle contractile function.

It is noticeable that decaffeinated coffee had a similar effect as regular coffee in stimulating smooth muscle contractions and improving intestinal motility in POI. According to the manufacturer, more than 97% of the caffeine is removed in their decaffeinated coffee. These data suggest that caffeine is not the main component responsible for the beneficial effect of coffee in POI. Coffee is a complex dietary matrix. There are hundreds of biologically active components in a brewed coffee solution, and it is difficult to determine which component(s) is (are) responsible for the benefits in POI. Among the many bioactive constituents in a coffee solution, we believe that melanoidins, polyphenols, or choline may represent the most likely candidates involved in the effects on smooth muscle contractions. Melanoidins such as Arg-Glu were found to induce gastric muscle contractions [30]. There have been reports that polyphenols such as chlorogenic acid (CGA) and caffeic acid (CA) have protective effects on the enteric nervous system (ENS) [31,32]. However, CA seems to lead to smooth muscle relaxation [33]. Finally, choline, as an essential human nutrient, is contained in brewed coffee, though in small amounts [34]. Choline is known to stimulate contractions of gut smooth muscle [35]. We found that the effective concentrations of choline to induce mouse intestinal contractions are about 10 mg/mL (unpublished observations, XZ Shi et al.). Considering that the concentration of choline in brewed coffee is only around 6.24 mg/8 OZ (0.026 mg/mL) [34], it is unlikely that choline is the sole substance responsible for the contraction-stimulating effect of coffee. Further investigation is warranted to determine the responsible substance(s) for the beneficial effect of coffee in POI. Such studies may help to better understand the health benefits of coffee and possibly identify pharmacological targets for the management of POI and other motility disorders.

To better understand the mechanisms underlying coffee’s action on smooth muscle contractility, we compared the direct contractile property of coffee on smooth muscle strips in vitro in the absence and presence of tetrodotoxin (TTX), hexamethonium (Hex), and atropine (Atr) [15,22]. The neurotransmitter acetylcholine (ACh) is released from excitatory motor neurons in the intrinsic myenteric plexus of the ENS and acts on muscarinic receptors in gut smooth muscle cells, thereby facilitating neural control of gut smooth muscle contractions. On the other hand, ACh also interacts with nicotinic receptors in enteric motor neurons, allowing the extrinsic nervous system (i.e., the parasympathetic nervous system) to innervate the myenteric ganglia and indirectly regulate smooth muscle contractions. Thus, the neural control of gut smooth muscle contractions involves pre-ganglia neural innervation via nicotinic receptors acting on the motor neurons in the myenteric ganglia. The excitatory motor neurons are primarily cholinergic, acting on cholinergic muscarinic receptors in gut smooth muscle cells, leading to contractions [22,36]. In our study, pretreatment with neural blocker TTX or nicotinic receptor antagonist Hex did not have any inhibitory effect on coffee-evoked contractile response, suggesting that coffee does not act on pre-ganglia or post-ganglia neural transmission. However, the cholinergic muscarinic receptor antagonist atropine completely abolished coffee-evoked contractions in the muscle strips. This data suggests that coffee exerts its contractile effect through a mechanism involving cholinergic muscarinic receptors on smooth muscle cells. Further studies are warranted to determine the precise molecular target(s) of coffee in evoking muscarinic receptor-dependent contractions in intestinal smooth muscle.

There are some limitations of the study. First, we used only a single time point (24 h) to assess the effects of coffee on inflammation and motility in the model. It was found that coffee treatment did not reduce inflammation but improved motility at the 24 h time point. Given that POI is a dynamic process, whether coffee influences earlier or later inflammatory responses may deserve further investigation. Second, we studied four representative inflammatory markers (IL-1beta, IL-6, CCL2, and CXCL-1), which were all significantly increased in the model. A broader inclusion of inflammatory characterization may help us to better understand the effect of coffee on the model. In the in vivo studies, we aimed to allocate five or six animals in each group (including three males and two or three females). Preliminary observation was that the differences between the two sexes in inflammation score, gene expression, and muscle contractility are minor. However, due to the modest sample sizes, we could not conduct a sex-stratified analysis.

## 5. Conclusions

Taken together, coffee consumption does not attenuate inflammatory response at the 24 h time point in mouse POI. However, coffee significantly improves GI motor function in ileus by stimulating smooth muscle contractions and accelerating GI transit. Coffee stimulates gut smooth muscle contractions through a cholinergic muscarinic receptor-dependent mechanism in a caffeine-independent manner.

## Data Availability

The data that support the findings of the study are available from the corresponding author upon reasonable request.

## Abbreviations

ACh, acetylcholine; Atr, atropine; CCL2, C-C motif chemokine ligand 2; CXCL-1, C-X-C motif chemokine ligand 1; Decaf, decaffeinated coffee; ENS, enteric nervous system; GI, gastrointestinal; GC, geometric center; Hex, hexamethonium; IL-1, interleukin 1; IL-6, interleukin 6; IM, intestinal manipulation; POI, postoperative ileus; qRT-PCR, quantitative reverse transcription polymerase chain reaction; SMC, smooth muscle cell; TTX, tetrodotoxin.
